# First Report and Biological Characterization of *Penicillium crustosum* Causing Root Rot in *Polygonatum kingianum* (Yunnan, China)

**DOI:** 10.3390/plants15111739

**Published:** 2026-06-03

**Authors:** Ming-Xian Zhang, Zi-Han Chen, Li-Hua Wang, Xiao-Yi Yang, You-Yong Zhu, Yu Zhao

**Affiliations:** 1 Department of Plant Protection, Yunnan Agricultural University, Kunming 650201, China; 18083416982@163.com (M.-X.Z.);; 2 State Key Laboratory for Conservation and Utilization of Bio-Resources in Yunnan, Yunnan Agricultural University, Kunming 650504, China

**Keywords:** biological characteristics, pathogen identification, *Penicillium crustosum*, *Polygonatum kingianum*, root rot

## Abstract

Root rot is a major disease restricting the cultivation and production of *Polygonatum kingianum* Coll. et Hemsl. This study aimed to identify the causal agent and characterize its biological properties. Pathogens were isolated from diseased rhizomes showing typical symptoms, and their pathogenicity was confirmed through Koch’s postulates using both detached rhizome inoculation and field pot experiments with spore suspension irrigation, in which typical root rot symptoms were reproduced. Based on morphological characteristics and multi-locus phylogenetic analysis (*ITS*, *CaM*, *RPB2*, and *TUB*), the pathogen was identified as *Penicillium crustosum*. Biological characterization revealed that the optimal conditions for mycelial growth and sporulation were 25 °C and pH 8–9, with Czapek agar being the most suitable medium. Light conditions significantly influenced fungal development; continuous darkness (24 h) favored mycelial growth, while an alternating light/dark cycle (12 h/12 h) significantly enhanced sporulation. Furthermore, the pathogen exhibited the highest utilization efficiency for soluble starch as a carbon source and peptone or yeast extract as a nitrogen source. These physiological traits suggest a strong adaptive capacity of the pathogen to environmental conditions associated with host rhizomes, which may contribute to disease development under cultivation conditions. To our knowledge, this is the first report of *P. crustosum* causing root rot in *P. kingianum*. The findings provide a basis for accurate pathogen identification and improve current understanding of the biological characteristics of this pathogen, thereby supporting future studies on disease monitoring and management.

## 1. Introduction

*Polygonatum kingianum* Coll. et Hemsl. is a well-known medicinal and edible plant widely cultivated in southwestern China [1]. It is a perennial herb with thick, fleshy, subcylindrical or submoniliform rhizomes as the main medicinal organ, and prefers shady, moist habitats such as forests, thickets, or damp grassy slopes at an altitude of 700–3600 m [2]. Its rhizomes are rich in polysaccharides, saponins, and flavonoids and exhibit diverse pharmacological activities [3,4,5]. With the rapid development of the health food and traditional medicine industries, the cultivation area of *P. kingianum* has expanded substantially in recent years. However, continuous cropping and intensive cultivation practices have led to increasingly severe soil-borne diseases, posing a major constraint to sustainable production [6,7].

Root rot is one of the most destructive diseases affecting *P. kingianum*, characterized by rhizome decay and the subsequent wilting and death of aboveground tissues, resulting in significant yield and quality losses [8,9]. Previous studies have shown that root rot of *Polygonatum* species is mainly caused by typical soil-borne pathogens, including *Fusarium* spp., *Colletotrichum* spp., and *Aspergillus* spp. [8,10,11]. Disease development is also closely related to soil microecology and environmental conditions [12]. Nevertheless, the diversity of fungal species associated with diseased rhizomes under field cultivation remains insufficiently understood [13,14,15]. In particular, whether unreported or overlooked pathogenic fungi contribute to disease occurrence remains unclear, which limits accurate diagnosis and targeted disease management.

Based on this knowledge gap, we hypothesized that root rot of *P. kingianum* may involve new or previously overlooked pathogenic fungi, and that the dominant pathogen may possess biological traits associated with environmental adaptation and pathogenicity. Therefore, the objectives of this study were to: (1) isolate and identify the pathogenic fungi associated with root rot using morphological observation and multi-locus phylogenetic analysis; (2) verify pathogenicity following Koch’s postulates; and (3) characterize the biological properties of the dominant pathogen. This study aims to clarify the etiology of root rot in *P. kingianum*, expand current knowledge of pathogen diversity associated with this disease, and provide a basis for early diagnosis, accurate identification, and sustainable disease management in field production.

## 2. Results

### 2.1. Disease Symptoms in the Field

The disease primarily affected the underground rhizomes of *Polygonatum kingianum* Field investigation results showed that the natural disease incidence of root rot reached 20.5% in the surveyed planting area, indicating that this disease had become prevalent in local cultivation.

**Figure 1:**
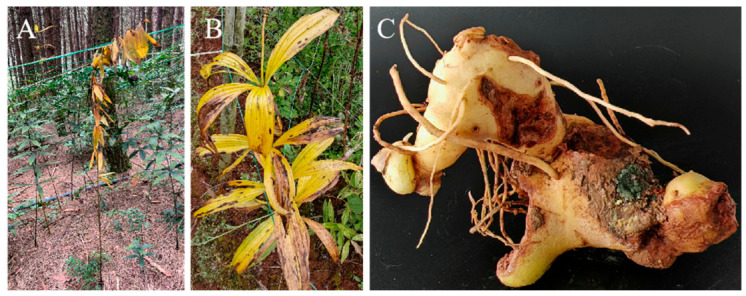
Field symptoms of naturally infected *Polygonatum kingianum* (**A**,**B**) Field symptoms of naturally infected above-ground parts of *P. kingianum* and (**C**) field symptoms of naturally infected underground parts of *P. kingianum.*

In the field, diseased plants often exhibited general decline symptoms in the aboveground parts, including leaf chlorosis, necrosis, curling, and drooping (Figure 1A,B). These symptoms were typically observed in plants with severely decayed rhizomes. Infected rhizomes initially developed dark brown to black necrotic lesions on the epidermis. As the disease progressed, the lesions expanded and penetrated into internal tissues, leading to tissue softening and collapse. Under humid conditions, dense dark green mold growth was frequently observed on the surface of decayed rhizomes (Figure 1C). Pathogenicity tests were subsequently conducted to confirm the causal pathogen.

### 2.2. Pathogen Identification and Pathogenicity Analysis

A total of 36 fungal strains were isolated and purified from diseased rhizome samples of *Polygonatum kingianum* (Figure S1). All 36 isolates were subjected to an in vitro pathogenicity test following Koch’s postulates, and disease occurrence was investigated 7 days after inoculation. The results showed that 16 of the 36 strains were pathogenic to *P. kingianum*, while the remaining 20 strains were non-pathogenic and were excluded from subsequent studies (Figures S2 and S3).

*ITS* sequence amplification and sequencing were performed on the 16 pathogenic strains, and the obtained sequences were aligned with available data in the NCBI database. A phylogenetic tree was constructed based on *ITS* sequences using MEGA 11 software (Figure S4). The results indicated that the 16 pathogenic strains belonged to five species within three genera: three isolates of *Colletotrichum* sp. (Hcu51, Hcu52, Hcu53), four isolates of *Fusarium oxysporum* (W1, W2, W6, W14), three isolates of *Fusarium equiseti* (W7, W9, W12), one isolate of *Fusarium solani* (W13), and five isolates of *Penicillium crustosum* (LTD0-3, LTD0-9, LTD0-10, LTD0-15, LTD0-16).

**Figure 2:**
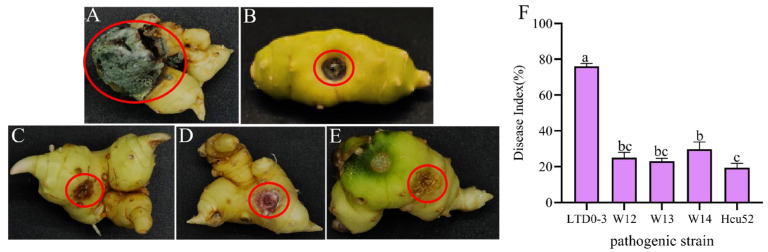
Pathogenicity assays of different pathogens causing *Polygonatum kingianum* root rot. (**A**) Symptoms induced by strain LTD0-3; (**B**) symptoms induced by strain W12; (**C**) symptoms induced by strain W13; (**D**) symptoms induced by strain W14; (**E**) symptoms induced by strain Hcu52-2; (**F**) disease index of *P. kingianum* root rot after inoculation with different strains. Red circles indicate lesion positions on rhizomes induced by tested strains. The different lowercase letters indicate significant differences (*p* < 0.05).

To clarify the pathogenic differences among different species, one representative strain was selected from each of the five species, including LTD0-3 (*P. crustosum*), W12 (*F. equiseti*), W13 (*F. solani*), W14 (*F. oxysporum*), and Hcu52 (*Colletotrichum* sp.). Pathogenicity assays confirmed that all five representative strains could infect *P. kingianum* and cause typical root rot symptoms (Figure 2A–E). Virulence analysis showed that strain LTD0-3 exhibited the highest pathogenicity. It induced rapid soft rot of host tissues and formed a characteristic blue–green mycelial layer at the inoculation site (Figure 2A). Disease index analysis (Figure 2F) revealed that the disease index of LTD0-3 reached 76%, which was significantly higher than those of the other representative strains (W12: 25%, W13: 23%, W14: 29%, Hcu52: 19%). Considering its strongest virulence and highest field isolation frequency, strain LTD0-3 was selected as the target strain for subsequent biological characterization.

### 2.3. Pathogenicity Verification by In Vitro and In Vivo Inoculation

**Figure 3:**
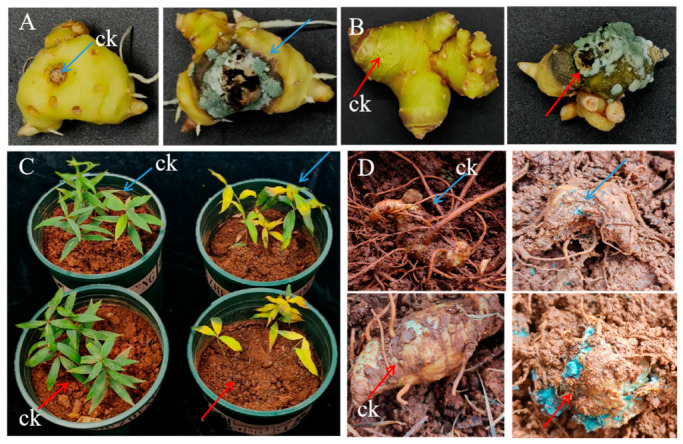
Pathogenic characteristics of the pathogenic strain LTD0-3 on *Polygonatum kingianum*. (**A**) Disease symptoms after non-wound inoculation with strain LTD0-3 in vitro; (**B**) disease symptoms after wound inoculation with strain LTD0-3 in vitro; (**C**) disease symptoms on above-ground parts after root drenching with the spore suspension of pathogenic strain LTD0-3; (**D**) disease symptoms on underground rhizomes after root drenching with the spore suspension of pathogenic strain LTD0-3; (**A**,**B**) ck indicates the control group inoculated with blank PDA blocks; (**C**,**D**) ck indicates the control group treated with sterile water; (**A**–**D**) blue arrows indicate the non-wound root drenching group, and red arrows indicate the wound root drenching group.

In vitro inoculation results showed that all *Polygonatum kingianum* rhizomes developed disease symptoms after inoculation with strain LTD0-3, with water-soaked, irregularly margined brown lesions appearing at the inoculation sites (Figure 3A,B). The lesion expansion rate in the wounded inoculation treatment (Figure 3B) was faster than that in the unwounded inoculation (Figure 3A), indicating that wounds facilitate pathogen infection. At 14 days after inoculation, the infected tissues were obviously rotten, with a dark green mycelial layer formed on the surface, whereas the control tubers remained healthy and asymptomatic throughout.

The pathogenicity was further verified by in vivo root-drench inoculation. The results demonstrated that potted seedlings of *P. kingianum* treated with spore suspension of strain LTD0-3 showed typical root rot symptoms: yellowing and wilting of above-ground leaves (Figure 3C) and soft rot of underground rhizome tissues with visible blue–green mycelial layer(Figure 3D). The disease severity in the wounded root-drench group was significantly higher than that in the unwounded group, while the control plants and rhizomes remained healthy and symptomless (Figure 3C,D).

### 2.4. Morphological and Molecular Identification

**Figure 4:**
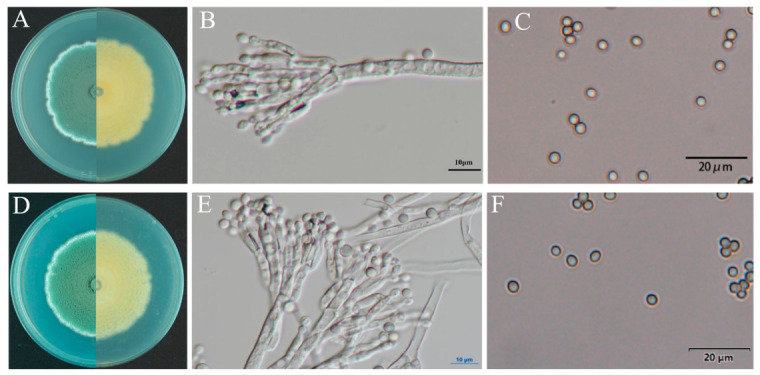
Morphological characteristics of *Penicillium crustosum* strain LTD0-3 cultured on PDA medium. (**A**) Front (left) and reverse (right) views of the LTD0-3 colony; (**B**) hyphal morphology of the pathogenic fungus, scale bar = 10 µm; (**C**) conidial morphology of the pathogenic fungus, scale bar = 20 µm; (**D**–**F**) morphology of colonies, hyphae and conidia of the pathogen re-isolated from diseased *P. kingianum* rhizomes after inoculation; (**E**) = 10 µm; (**F**) = 20 µm.

Strain LTD0-3 was cultured on potato dextrose agar (PDA) plates for morphological observation. Colonies were initially white and floccose, gradually turning grayish green to dark green with age. The colony surface appeared floccose to powdery, with abundant development of conidial layers. Colonies were compact in texture with relatively regular margins, which were often surrounded by a narrow white mycelial zone. The reverse side of the colonies was yellow to yellowish brown, and small amounts of transparent to pale yellow exudate droplets were occasionally observed under certain culture conditions. Sclerotia were not observed (Figure 4A).

Microscopic examination revealed that the hyphae were hyaline, septate, and branched. Conidiophores arose erectly from the vegetative hyphae, with smooth to slightly roughened walls, and were often curved. The apices of conidiophores were swollen and formed typical penicillate (brush-like) branching structures (Figure 4B). Conidia were produced in chains, nearly globose to subglobose, light green in color, with smooth to slightly roughened surfaces, and measured approximately 3–5 μm in diameter (Figure 4C). The colony characteristics and microscopic morphological features of strain LTD0-3 were consistent with those described for *Penicillium crustosum*.

The pathogen was re-isolated from the lesions of diseased rhizomes and subcultured. The morphological characteristics of mycelia and conidia of the re-isolated strain were consistent with those of the inoculated strain LTD0-3 (Figure 4D–F).

### 2.5. Molecular Identification of the Pathogen

Genomic DNA was extracted from strain LTD0-3, and the *ITS*, *CaM*, *TUB*, and *RPB2* regions were amplified using the corresponding primer pairs. The obtained sequences were subjected to homology searches against the NCBI GenBank database. Sequence similarity analysis showed that strain LTD0-3 shared 99.65%, 100%, 99.36%, and 100% identity with reference strains of *Penicillium crustosum* based on the *ITS*, *CaM*, *TUB*, and *RPB2* loci, respectively.

**Figure 5:**
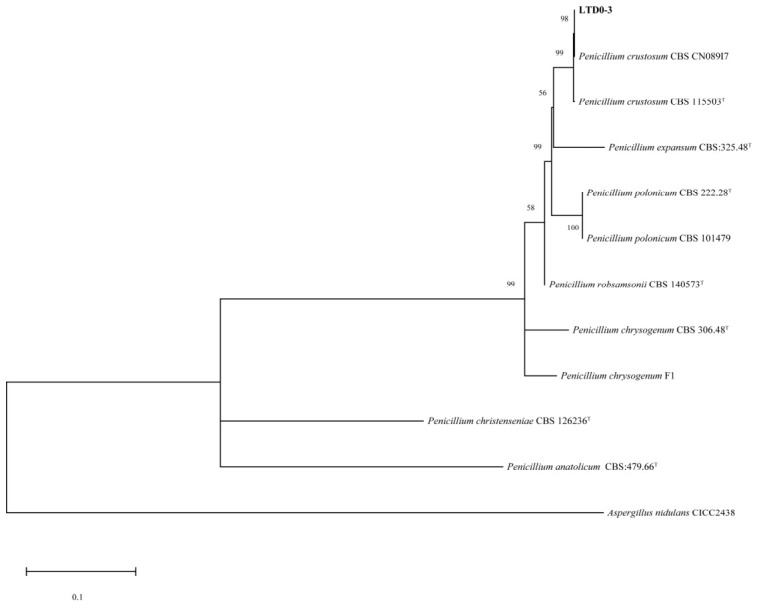
The phylogenetic tree of strain LTD0-3 was constructed using the maximum likelihood method. *Aspergillus nidulans* CICC2438 was used as the outgroup, and bootstrap values (1000 replicates) are shown at the branch points. The scale bar indicates 0.05 nucleotide substitutions per nucleotide position. T = type strain.

Phylogenetic analysis was performed using the combined *ITS*, *CaM*, *TUB*, and *RPB2* sequence dataset. The sequences were aligned using ClustalW in MEGA version 11.0, and the maximum likelihood tree was constructed under the GTR + G + I nucleotide substitution model. Bootstrap analysis was conducted with 1000 replicates to evaluate branch support. In the resulting phylogenetic tree, strain LTD0-3 clustered within the P. crustosum clade with strong phylogenetic support, showing a bootstrap value of 99% (Figure 5). Combined with the morphological characteristics, the molecular evidence confirmed that strain LTD0-3 was identified as *P. crustosum*.

The nucleotide sequences of strain LTD0-3 were deposited in the GenBank database under accession numbers PX717000 (*ITS*), PX733343 (*TUB*), PZ459949 (*CaM*), and PZ459950 (*RPB2*).

### 2.6. Effects of Temperature and Light Conditions on Colony Growth and Sporulation

**Figure 6:**
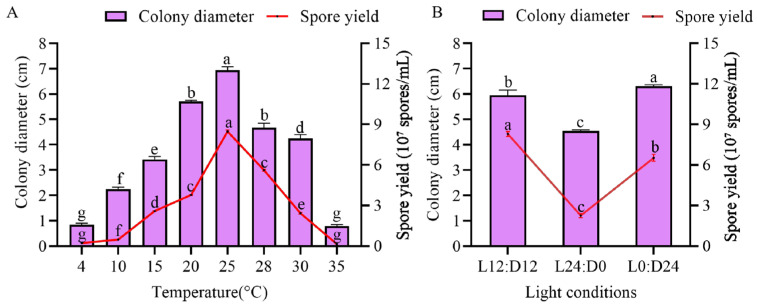
The effects of different temperatures and light conditions on colony diameter and sporulation. Different lowercase letters indicate significant differences between groups in terms of colony diameter and spore production (*p* < 0.05). (**A**) The effect of different temperatures on colony diameter and sporulation and (**B**) the effects of different light conditions on colony diameter and sporulation. L12:D12, 12 h light/12 h dark cycle; L24:D0, 24 h continuous light; L0:D24, 24 h continuous darkness.

*Penicillium crustosum* was able to grow over a temperature range of 4–35 °C, and both colony growth and sporulation differed significantly among the tested temperatures. Colony growth and spore production were markedly reduced at 4 °C and 35 °C. With increasing temperature, colony diameter and sporulation gradually increased, reaching their highest values at 25 °C, with a mean colony diameter of 6.94 cm and a spore concentration of 8.48 × 10^7^ spores/mL. When the temperature exceeded 28 °C, both colony growth and sporulation declined significantly (Figure 6A).

Light conditions also had a significant effect on the colony growth and sporulation of *P. crustosum.* The fungus was able to grow under all tested light regimes, including a 12 h light/12 h dark cycle, continuous light (24 h light), and continuous darkness (24 h dark). The largest colony diameter (6.32 cm) was observed under continuous darkness, followed by the 12 h light/12 h dark regime (5.95 cm), whereas continuous light resulted in the smallest colony diameter (4.54 cm). In contrast, sporulation was highest under the 12 h light/12 h dark regime, reaching 8.29 × 10^7^ spores/mL, while the lowest spore production (2.26 × 10^7^ spores/mL) was recorded under continuous light conditions (Figure 6B).

### 2.7. Effects of pH on Colony Growth and Sporulation

**Figure 7:**
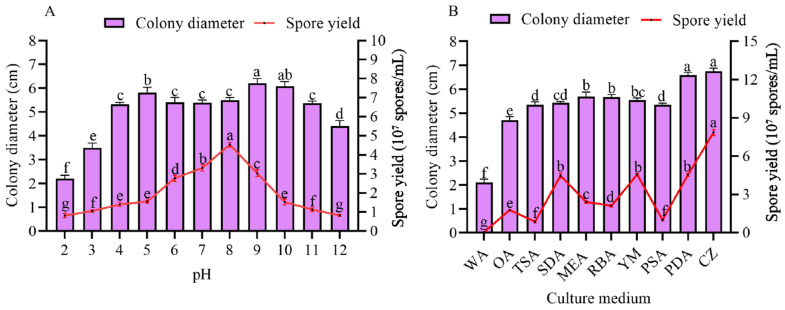
Effects of different pH and culture media on colony diameter and sporulation. Different lowercase letters indicate significant differences between groups in terms of colony diameter and spore production (*p* < 0.05). (**A**) The effect of different pH on colony diameter and sporulation, and (**B**) the effect of different culture media on colony diameter and sporulation. WA: Water agar medium; OA: oatmeal agar medium; TSA: tryptic soy agar medium; SDA: Sabouraud’s dextrose agar medium; MEA: malt extract agar medium; RBA: Rose Bengal agar medium; YM: yeast malt extract medium; PSA: potato sucrose agar medium; PDA: potato dextrose agar medium; CZ: Czapek medium.

*Penicillium crustosum* exhibited growth and sporulation over a wide pH range from 2 to 12. Colony growth and spore production varied significantly among the tested pH conditions. The largest colony diameter (6.21 cm) was observed at pH 9, whereas the highest spore concentration (4.54 × 10^7^ spores/mL) was recorded at pH 8 (Figure 7A).

### 2.8. Effects of Culture Media on Colony Growth and Sporulation

Significant differences in the colony growth of *Penicillium crustosum* were observed among the tested culture media. Colony growth was markedly inhibited on water agar (WA), whereas the pathogen was able to grow normally on the remaining nine media. The largest colony diameters were recorded on potato dextrose agar (PDA) and Czapek agar, measuring 6.58 cm and 6.74 cm, respectively, with no significant difference between the two media (*p* > 0.05). In addition, sporulation was highest on Czapek agar, reaching 7.85 × 10^7^ spores/mL, which was significantly greater than that observed on the other tested media (Figure 7B).

### 2.9. Effects of Carbon and Nitrogen Sources on Colony Growth and Sporulation

**Figure 8:**
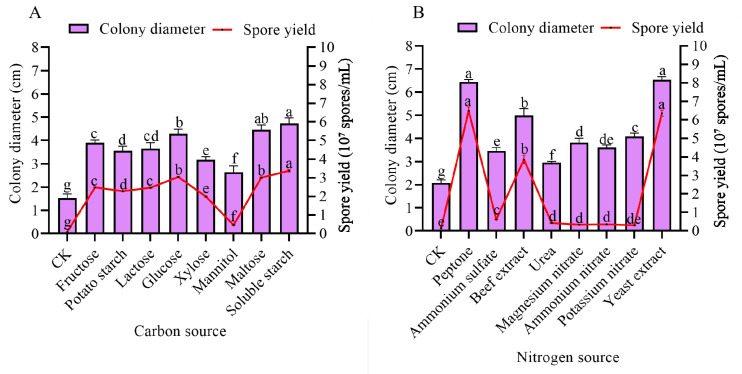
The effects of different carbon and nitrogen sources on colony diameter and sporulation. Different lowercase letters indicate significant differences between groups in terms of colony diameter and spore production (*p* < 0.05). (**A**) The effect of different carbon sources on colony diameter and sporulation and (**B**) the effect of different nitrogen sources on colony diameter and sporulation.

Colony growth of *Penicillium crustosum* was significantly reduced on Czapek medium without an added carbon source (CK), with a mean colony diameter of only 1.53 cm. Supplementation with different carbon sources significantly enhanced fungal growth. Among the tested carbon sources, soluble starch and maltose supported the largest colony diameters, reaching 4.73 cm and 4.46 cm, respectively. Sporulation was also highest when soluble starch was supplied as the sole carbon source, with a spore concentration of 3.37 × 10^7^ spores/mL. In contrast, mannitol resulted in the smallest colony diameter (2.64 cm) and the lowest spore production (0.46 × 10^7^ spores/mL) among the tested carbon sources (Figure 8A).

*P. crustosum* was able to grow on Czapek media containing all tested nitrogen sources. The highest colony growth and sporulation were observed when peptone or yeast extract was used as the nitrogen source. Specifically, when peptone was supplied, the colony diameter and spore concentration reached 6.44 cm and 6.49 × 10^7^ spores/mL, respectively, while yeast extract supported similar levels, with values of 6.54 cm and 6.36 × 10^7^ spores/mL. Beef extract resulted in moderate colony growth and sporulation (4.99 cm and 3.86 × 10^7^ spores/mL), whereas urea led to the lowest colony diameter (2.96 cm) and spore production (0.43 × 10^7^ spores/mL) among the tested nitrogen sources (Figure 8B).

## 3. Discussion

In this study, *Penicillium crustosum* was isolated from diseased rhizomes of *Polygonatum Kingianum*, and its pathogenicity was supported by inoculation assays, quantitative disease assessment, and pathogen re-isolation from symptomatic tissues. Field investigation showed a natural disease incidence of 20.5% in the plantation surveyed. Among the isolates obtained from diseased rhizomes, strain LTD0-3 showed strong pathogenicity, with a disease index of 76%, and reproduced typical root rot symptoms in both detached rhizome and potted plant inoculation assays. The same fungus was re-isolated from symptomatic tissues, fulfilling Koch’s postulates. To our knowledge, this is the first report of *P. crustosum* causing root rot of *P. kingianum*.

Species of *Penicillium* are more commonly reported as saprophytes or postharvest-associated fungi. *P. crustosum* has been widely recognized as a postharvest pathogen causing decay of pome fruits, citrus, and nuts [16,17,18]. The present study extends the known disease association of this species to rhizome rot of a medicinal and edible crop. However, root rot of *P. kingianum* may involve multiple fungal taxa [7]. Therefore, although *P. crustosum* showed strong pathogenicity in this study, its relative importance in field disease development requires further investigation.

Environmental adaptability plays a key role in pathogen establishment and disease development [19,20]. In this study, strain LTD0-3 grew over a broad temperature range of 4–35 °C, with optimal mycelial growth and sporulation at 25 °C. This optimum was close to the annual mean soil temperature at the sampling site in Lancang County, Yunnan Province (25.7 °C), suggesting that local soil temperature may provide favorable conditions for pathogen growth and reproduction during cultivation. The broad temperature tolerance of LTD0-3, together with its ability to grow at 4 °C, indicates tolerance to low-temperature conditions and suggests that the pathogen may persist under diverse environmental conditions. These characteristics may pose potential risks during both field cultivation and postharvest storage, particularly when temperature management is inadequate [21]. LTD0-3 also showed broad pH adaptability, growing and sporulating at pH 2–12. Although the soil pH at the sampling site was slightly acidic (5.26–6.51), it remained within the tolerance range of the strain, suggesting that the pathogen can persist under local soil conditions. Light conditions also affected fungal development. Continuous darkness promoted mycelial growth, which is ecologically relevant because *P. kingianum* rhizomes are buried in soil and exposed to near-dark conditions. In contrast, continuous light is unlikely to represent the natural soil environment and should be considered mainly as an experimental condition. Enhanced sporulation under a 12 h light/12 h dark cycle may be associated with exposed diseased rhizomes, plant residues, harvesting, handling, or storage [22,23]. Together, these results suggest that LTD0-3 has strong environmental adaptability, although the roles of temperature, pH, and light in field disease development require further investigations.

The nutritional utilization pattern of *P. crustosum* may also be related to its colonization of *P. kingianum* rhizomes. The pathogen showed strong growth and sporulation when soluble starch was supplied as the carbon source, and maltose also supported relatively high mycelial growth. This preference is consistent with the high polysaccharide and starch content of *P. kingianum* rhizomes [3]. Species of *Penicillium* are known to produce extracellular enzymes involved in the degradation of plant-derived carbohydrates, which may facilitate the colonization and decomposition of host tissues [24]. In addition, the ability of LTD0-3 to utilize several organic nitrogen sources suggests metabolic flexibility, although its ecological relevance in soil and rhizosphere environments remains to be clarified. Together with its broad pH tolerance, this nutritional adaptability may contribute to the persistence of *P. crustosum* in host-associated and soil habitats [25]. However, these biological traits were determined under controlled in vitro conditions, and their actual roles in disease development and pathogen survival under field conditions require further investigation.

Another point deserving attention is the potential relevance of *P. crustosum* contamination in medicinal and edible crops. Previous studies have shown that some strains of *P.crustosum* are capable of producing secondary metabolites such as penitrem A and roquefortine C [26]. Given that *P. kingianum* is widely used as a medicinal and edible crop in traditional Chinese medicine formulas and functional foods, its contamination by toxigenic *Penicillium* may represent a potential safety concern [27].

Overall, this study demonstrates that *P. crustosum* is associated with the root rot of *P. kingianum* and provides new insights into the biological characteristics of this pathogen. The strong pathogenicity of strain LTD0-3, its relatively high isolation frequency, and its adaptability to a broad range of temperature and pH conditions suggest that this fungus may play an important role in disease occurrence under suitable environmental conditions. Although the present study did not investigate field epidemiology, natural infection processes, or management strategies, the findings provide a basis for future research on pathogen ecology, infection biology, rhizosphere interactions, mycotoxin risk, and disease management in *P. kingianum* cultivation systems.

## 4. Materials and Methods

### 4.1. Plant Material and Sample Collection

In July 2025, rhizomes of *Polygonatum kingianum* showing typical root rot symptoms were collected from an understory medicinal plant plantation in Lancang County, Pu’er City, Yunnan Province, China (102°10′ E, 25°23′ N; altitude approximately 1800 m). The average annual soil temperature at the sampling site was 25.7 °C, and the field soil pH ranged from 5.26 to 6.51, indicating a slightly acidic soil environment. Samples were transported to the laboratory for pathogen isolation and identification.

A field disease survey was simultaneously carried out in the plantation. A total of 200 *P. kingianum* plants were randomly investigated, and the field natural disease incidence was calculated according to the field survey data.

### 4.2. Isolation and Purification of the Pathogen

Pathogens were isolated using the tissue isolation method [28]. Small tissue segments (3 mm × 3 mm) were excised from the boundary between diseased and healthy tissues of infected rhizomes of *Polygonatum kingianum.* The surface of the segments was sterilized with 75% ethanol for 30 s, followed by 1% sodium hypochlorite for 1 min, rinsed three times with sterile distilled water, and placed on potato dextrose agar (PDA). Plates were incubated at 25 °C, and single colonies with distinct morphologies were subcultured repeatedly to obtain pure isolates. Purified cultures were stored on PDA slants at −20 °C in a refrigerator (Haier Co., Ltd., Qingdao, Shandong, China) for further use.

### 4.3. Pathogenicity Test

Pathogenicity was determined following Koch’s postulates [29], with *Polygonatum kingianum* as the host. Two inoculation methods were used: in vitro and root drenching (in vivo), each with three treatments (prick-wound, non-wound, and blank control) [30,31]. All 36 fungal isolates obtained from diseased rhizomes were first subjected to in vitro pathogenicity screening. Isolates causing typical root rot symptoms were regarded as pathogenic, while non-pathogenic strains were eliminated from subsequent tests. One representative strain was selected from each pathogenic species for further virulence comparison.

Healthy *P. kingianum* rhizomes were selected, their surfaces sterilized with 75% ethanol for 30 s, thoroughly rinsed with sterile water, and then air-dried naturally. For the wound group, 3–5 pricks were made on the rhizome surface using a sterile needle, while no wounding treatment was performed on the non-wound group. Mycelial plugs (5 mm in diameter) from 7-day-old PDA cultures were inoculated onto the rhizomes, and sterile agar plugs were used as the blank control. The rhizomes were incubated at 25 °C under moist conditions for 14 days, with three replicates of nine rhizomes each. After incubation, the disease severity of each rhizome was investigated, and the disease index (DI) was calculated to evaluate the pathogenicity of different strains, thereby selecting/identifying the most virulent isolate.

Owing to the irregular and tuberous morphology of *P. kingianum* rhizomes, the lesion area and total rhizome area were measured using ImageJ 1.54 software. The lesion proportion was accurately calculated based on the scanned images, the disease severity was graded on a 0–4 scale as follows: Grade 0, no disease lesions; Grade 1, disease lesions accounting for <10% of the total rhizome area; Grade 2, disease lesions accounting for 10–33% of the total rhizome area; Grade 3, disease lesions accounting for 33–67% of the total rhizome area; Grade 4, disease lesions accounting for >67% of the total rhizome area [32]. The disease index was calculated using the formula:

$$DI=\;\frac{\sum (Si\;\times \;Xi)}{4\;\times \;N}\times 100$$

*Si* is the severity rating, *Xi* is the number of rhizomes with the corresponding severity rating, and *N* is the total number of roots in one sampling site [33].

The most pathogenic root rot strain was selected to prepare its conidial suspension, and the spore concentration was adjusted to 1 × 10^7^ spores/mL using a hemocytometer(Qiujing Biochemical Reagent and Instrument Co., Ltd., Shanghai, China). Uniform potted *P. kingianum* seedlings were divided into three groups: the wound group was pricked (3–5 × 0.5 cm deep) at the stem base before drenching with 200 mL suspension per plant; the non-wound group was drenched directly with 200 mL suspension; the control group was treated with an equal volume of sterile water. Drenching was conducted once every 7 days, for a total of 3 applications. Disease incidence was investigated 21 days after the final inoculation, and pathogens were re-isolated from symptomatic tissues and compared with the original strain to fulfill Koch’s postulates.

### 4.4. Morphological Identification

The purified pathogen was inoculated onto potato dextrose agar (PDA) medium and incubated at 25 °C in a constant-temperature incubator (Bluepard Instruments Co., Ltd., Shanghai, China) in the dark for 10 days. Colony morphology and color were observed, and hyphae were picked with an inoculation needle and mounted on slides. Hyphal characteristics, color, conidiophores, and conidia were examined under a light microscope(Olympus (Evident Corporation), Tokyo, Japan), and images were captured and recorded [34].

### 4.5. Molecular Identification

Genomic DNA of the pathogen was extracted using the CTAB method. The target strain was amplified by PCR using the universal primer pairs for the Internal Transcribed Spacer (*ITS*), Calmodulin (*CaM*), RNA polymerase II second largest subunit (*RPB2*), and β-tubulin (*TUB*) regions (Table 1). The qualified PCR products were sequenced at Tsingke Biotechnology Co., Ltd. (Kunming, China).

The obtained sequences were subjected to homology searches against the NCBI GenBank database, and reference sequences with high similarity were selected (Table 2). Multiple sequence alignment was performed using ClustalW 2.1 in MEGA 11.0. The phylogenetic tree was constructed using the maximum likelihood (ML) method with the GTR + G + I nucleotide substitution model. Bootstrap support values were calculated with 1000 replicates to assess the robustness of the tree topology. All sequences of the strains were deposited in GenBank to obtain the corresponding accession numbers.

### 4.6. Biological Characterization

To provide Supplementary Information on the in vitro biological traits of the pathogen, the effects of selected environmental and nutritional factors on mycelial growth and sporulation were evaluated under controlled laboratory conditions. This experiment evaluated the impacts of temperature (4–35 °C), light conditions (continuous darkness, continuous light, and 12 h light/12 h light–dark cycle at a light intensity of 3000 lx), pH (2–12), different culture media, and various carbon and nitrogen sources on mycelial growth and sporulation [39,40,41]. The colony diameter was measured using the cross–cross method by measuring the diameters in two perpendicular directions and calculating their average to ensure data accuracy. Spore production was quantitatively determined using a hemocytometer [42]. All treatments were replicated five times to minimize the experimental error.

The specific procedure for spore yield determination was as follows: the tested strain was inoculated onto PDA plates and incubated at 25 °C under constant temperature for 7 days. After abundant sporulation, spores were gently scraped from the colony surface using a sterile scraper and added to 20 mL of sterile water. The suspension was vortexed for 5 min, followed by ultrasonic treatment for 10 min in an ultrasonic cleaner (KQ3200DE, Kunshan Ultrasonic Instruments Co., Ltd., Kunshan, Jiangsu, China) to fully disperse the spores, followed by filtration through four layers of sterile gauze to remove mycelial fragments. The filtered spore suspension was then adjusted to a final volume of 20 mL. A 100-fold serial dilution was performed, and 10 μL of the diluted suspension was loaded into a 25 × 16 hemocytometer. After allowing the spores to settle completely for 2 min, the number of spores in five medium squares was counted under a microscope, and the spore concentration was calculated using the following formula. Each treatment was performed in five replicates.

$$\mathrm{Spores}/\mathrm{mL}\;=\frac{\mathrm{Total}\;\mathrm{count}\;\mathrm{in}\;5\;\mathrm{squares}}{5}\;\times \;25\;\times \;10^{4}\;\times \;\mathrm{Dilution}\;\mathrm{factor}$$

### 4.7. Data Analyses

Data were analyzed using one-way analysis of variance (ANOVA) in SPSS 26.0. The graphs were prepared using GraphPad Prism 9.5.

## 5. Conclusions

This study represents the first report of *Penicillium crustosum* as a causal agent of root rot in *Polygonatum kingianum* and systematically characterizes its biological properties. Pathogenicity tests confirmed its ability to induce root rot symptoms, and physiological experiments revealed that the pathogen can grow across a wide range of temperatures (4–35 °C) and pH conditions (pH 2–12). The fungus also showed relatively strong growth on starch-based carbon sources, which are abundant in *P. kingianum* rhizomes. These biological characteristics may contribute to its capacity to colonize host tissues under suitable conditions. In addition, the ability of the pathogen to grow at relatively low temperatures suggests that it may persist during storage. However, toxin production by the isolates was not evaluated in this study. Further research on the ecology, infection biology, and potential toxin production of *P. crustosum* will help improve the understanding of its role in the root rot of *P. kingianum*.

## Figures and Tables

**Table 1:** Primer sequences used for PCR amplification.

Gene	Primer	Primer 5′→3′	Reference
*ITS*	ITS1	TCCGTAGGTGAACCTGCGG	[35]
	ITS4	TCCTCCGCTTATTGATATGC	
*TUB*	BT-2F	AACATGCGTGAGATTGTAAGT	[36]
	BT-4R	TAGTGACCCTTGGCCCAGTTG	
*CaM*	cmdD*3*	GACTCCCTGACCGAAGAGCA	[37]
	cmdA*2*	GCCTCGCGGATCATCTCATC	
*RPB2*	5F	GAYGAYMWGGATCAYTTYGG	[38]
	7cR	CCCATRGCTTGTTTCCCAT	

**Table 2:** Gene sequences used to construct the phylogenetic tree of *Penicillium crustosum.* Note: LTD0-3 represents the pathogenic isolate obtained in this study; all other isolates are reference strains downloaded from GenBank; ^T^ = type strain.

Isolate Code	Species	GenBank Accession			
		*ITS*	*CaM*	*TUB*	*RPB2*
LTD0-3	*Penicillium crustosum*	PX717000	PZ459949	PX733343	PZ459950
CBS 126236^T^	*Penicillium christenseniae*	NR_166035.1	JN606373.1	JN606680.1	JN606624.1
CBS:325.48^T^	*Penicillium expansum*	-	DQ911134.1	JQ965099.1	JF417427.1
CBS 306.48^T^	*Penicillium chrysogenum*	NR_077145.1	JX996273.1	AY495981.1	JN121487.1
F1	*Penicillium chrysogenum*	PQ394668.1	-	-	-
CBS:222.28^T^	*Penicillium polonicum*	NR_103687.1	KU896848.1	MN969392.1	JN406609.1
CBS 101479	*Penicillium polonicum*	JN942718.1	PV180550.1	JN398145.1	JN985410.1
CBS:479.66^T^	*Penicillium anatolicum*	MH858864.1	JN606571.1	-	JN606593.1
CBS 140573^T^	*Penicillium robsamsonii*	NR_144866.1	-	-	-
CBS 115503	*Penicillium crustosum*	MH862985.1	-	MN969379.1	MN969114.1
CN089I7	*Penicillium crustosum*	-	OR241869.1	OR241810.1	-
CICC2438	*Aspergillus nidulans*	-	-	KM491313.1	-

## Data Availability

The nucleotide sequence data generated in this study have been deposited in the NCBI GenBank database under accession numbers PX717000 (*ITS*), PZ459949 (*CaM*), PX733343 (*TUB*), and PZ459950 (*RPB2*). All other data supporting the findings of this study are available from the corresponding author upon reasonable request.

## Supplementary Materials

The following supporting information can be downloaded at https://www.mdpi.com/article/10.3390/plants15111739/s1. Figure S1: Colony morphology of 36 fungal isolates causing root rot of *Polygonatum kingianum*; Figure S2: Disease symptoms of *Polygonatum kingianum* rhizomes after inoculation with root rot pathogens; Figure S3: Pathogenicity evaluation of root rot pathogens; Figure S4: Phylogenetic tree constructed using ITS sequences of pathogenic fungi causing root rot in *Polygonatum kingianum*. Note: The phylogenetic tree was constructed using the Maximum Likelihood (ML) method, with 1000 bootstrap replicates. Figure S5: Colony characteristics of the pathogen under diverse culture conditions.

## Abbreviations

The following abbreviations are used in this manuscript:

*ITS*	Internal Transcribed Spacer
*CaM*	Calmodulin
*RPB2*	RNA Polymerase II Second Largest Subunit
*TUB*	β-tubulin
WA	Water Agar Medium
OA	Oatmeal Agar Medium
TSA	Tryptic Soy Agar Medium
SDA	Sabouraud’s Dextrose Agar Medium
MEA	Malt Extract Agar Medium
RBA	Rose Bengal Agar Medium
YM	Yeast Malt Extract Medium
PSA	Potato Sucrose Agar Medium
PDA	Potato Dextrose Agar Medium
CZ	Czapek Medium

## References

[1] Xiao L., Xu H., Wu T., Xie Q., Wen R., Wang L., Su B., Zhang H. (2024). Metabolomic Diversity in *Polygonatum kingianum* Across Varieties and Growth Years. Molecules.

[2] Wang J.D., Guo X.Y., Tang X.X. (2025). Molecular Identification and Phylogenetic Analysis of *Polygonatum kingianum* with Different Floral Colors on the Basis of Chloroplast Genomes. BMC Plant Biol..

[3] Luo L., Qiu Y., Gong L., Wang W., Wen R. (2022). A Review of *Polygonatum* Mill. Genus: Its Taxonomy, Chemical Constituents, and Pharmacological Effect Due to Processing Changes. Molecules.

[4] Lin H., Wang W., Peng M., Kong Y., Zhang X., Wei X., Shang H. (2024). Pharmacological Properties of *Polygonatum* and Its Active Ingredients for the Prevention and Treatment of Cardiovascular Diseases. Chin. Med..

[5] Zhu M., Chen G., Li J., Yi C., Yuan Y., Liu W., Zhang X. (2025). The Antitumor Potential of *Polygonatum* spp.: A Narrative Review of Traditional Uses, Bioactive Metabolites, and Multi-Targeted Mechanisms. Front. Pharmacol..

[6] Shen W., Lin X., Gao N., Zhang H., Yin R., Shi W., Duan Z. (2008). Land Use Intensification Affects Soil Microbial Populations, Functional Diversity and Related Suppressiveness of Cucumber Fusarium Wilt in China’s Yangtze River Delta. Plant Soil.

[7] Pang Z., Mao X., Xia Y., Xiao J., Wang X., Xu P., Liu G. (2022). Multiomics Reveals the Effect of Root Rot on *Polygonati* Rhizome and Identifies Pathogens and Biocontrol Strain. Microbiol. Spectr..

[8] Zhang L., Li H., Yang Z., Dong X., Ji P., Dong J., Wang Y. (2021). Identification of Pathogens Causing Root and Rhizome Rot of *Polygonatum kingianum* in Yunnan. Acta Phytopathol. Sin..

[9] Han Y., Sun T., Tang Y., Yang M., Gao W., Wang L., Sui C. (2025). Root Rot in Medicinal Plants: A Review of Extensive Research Progress. Front. Plant Sci..

[10] Ma W., Guo L., Wang X., Wang T., Yan S., Jin H., Zhang Z., Yang B. (2021). Isolation and Identification of a New Pathogen Causing Rhizome Rot of *Polygonatum kingianum*. China J. Chin. Mater. Medica.

[11] Wu W., Hu Y., Li C., Chen Y., Huang Q., Wang L., Li J., Liu R. (2024). First Report of Root Rot on Rhizome of *Polygonatum kingianum* Caused by *Aspergillus awamori*. Plant Dis..

[12] Hossain Z., Hubbard M., Gan Y., Bainard L.D. (2021). Root Rot Alters the Root-Associated Microbiome of Field Pea in Commercial Crop Production Systems. Plant Soil.

[13] Liu C., Zhang L., Li H., Dong J., He X., Qiu B. (2023). First Report of *Penicillium subrubescens* Causing Root Rot of *Knoxia roxburghii* in China. Plant Dis..

[14] Wang P., Zhao N., Liang C., Li X., Li J., Yan H., Sun Z., Zhang L. (2024). First Report of *Penicillium cellarum* Causing Rot Disease on *Dioscorea polystachya* in China. Plant Dis..

[15] Strausbaugh C.A., Dugan F. (2017). A Novel *Penicillium* sp. Causes Rot in Stored Sugar Beet Roots in Idaho. Plant Dis..

[16] Louw J.P., Korsten L. (2015). Pathogenicity and Host Susceptibility of *Penicillium* spp. on Citrus. Plant Dis..

[17] Qiao L., Jiao Y., Li X., Zhang Y., Lu L., Zhang X., Liu X. (2023). Herbal Smoke Fumigation for Controlling *Penicillium crustosum* in Fresh Walnuts. Food Res. Int..

[18] Vico I., Gaskins V., Duduk N., Vasić M., Yu J.J., Peter K.A., Jurick W.M. (2014). First Report of *Penicillium crustosum* Causing Blue Mold on Stored Apple Fruit in Serbia. Plant Dis..

[19] Juroszek P., Von Tiedemann A. (2013). Climate Change and Potential Future Risks through Wheat Diseases: A Review. Eur. J. Plant Pathol..

[20] Chakraborty S., Newton A.C. (2011). Climate Change, Plant Diseases and Food Security: An Overview. Plant Pathol..

[21] Dukare A.S., Singh R.K., Jangra R.K., Bhushan B. (2022). Non-Fungicides-Based Promising Technologies for Managing Post-Production *Penicillium* Induced Spoilage in Horticultural Commodities: A Comprehensive Review. Food Rev. Int..

[22] Zheng Y., Xie Y., Xie Y., Yu S. (2021). Asexual Reproduction and Vegetative Growth of *Bionectria ochroleuca* in Response to Temperature and Photoperiod. Ecol. Evol..

[23] Nmom F.W., Amadi L.O., Ngerebara N.N. (2021). Influences of Light Regimes on Reproduction, Germination, Pigmentation, Pathogenesis and Overall Development of a Variety of Filamentous Fungi—A Review. AJOB.

[24] Pacheco K.C.A., Barbosa-Tessmann I.P. (2026). Proteomic Profiling of Amylases Secreted by *Penicillium crustosum* UEM-45 and Characterization of Two Major Enzymes. 3 Biotech.

[25] Prusky D., McEvoy J.L., Saftner R., Conway W.S., Jones R. (2004). Relationship Between Host Acidification and Virulence of *Penicillium* Spp. on Apple and Citrus Fruit. Phytopathology.

[26] Rundberget T., Skaar I., Flåøyen A. (2004). The Presence of *Penicillium* and *Penicillium* Mycotoxins in Food Wastes. Int. J. Food Microbiol..

[27] Chen L., Wu J., Zhang S., Liu X., Zhao M., Guo W., Zhang J., Chen W., Liu Z., Deng M. (2025). Occurrence and Diversity of Fungi and Their Mycotoxin Production in Common Edible and Medicinal Substances from China. JoF.

[28] Naji Abdulrahman D., Haleem R.A. (2023). Morphological and Molecular Characterization of *Neoscytalidium* Isolates That Cause Canker and Dieback in *Eucalyptus* and Chinaberry Trees in Iraq. Plant Prot. Sci..

[29] Bhunjun C.S., Phillips A.J.L., Jayawardena R.S., Promputtha I., Hyde K.D. (2021). Importance of Molecular Data to Identify Fungal Plant Pathogens and Guidelines for Pathogenicity Testing Based on Koch’s Postulates. Pathogens.

[30] Kuang Y., Chen Q., Abah F., Su J., Yang Y., Yang Q., Ye Z., Wang Z., Chen M., Hu H. (2026). Identification and Biological Characterizations of the Causal Agent of Leaf Spot Disease in *Pseudostellaria heterophylla*. Plants.

[31] Su J., Wang J., Tang J., Yu W., Liu J., Dong X., Dong J., Chai X., Ji P., Zhang L. (2024). Zinc Finger Transcription Factor ZFP1 Is Associated with Growth, Conidiation, Osmoregulation, and Virulence in the *Polygonatum kingianum* Pathogen Fusarium Oxysporum. Sci. Rep..

[32] Bi Y.-M., Zhang X.-M., Jiao X.-L., Li J.-F., Peng N., Tian G.-L., Wang Y., Gao W.-W. (2023). The Relationship between Shifts in the Rhizosphere Microbial Community and Root Rot Disease in a Continuous Cropping American Ginseng System. Front. Microbiol..

[33] Jiao X.-L., Zhang X.-S., Lu X.-H., Qin R., Bi Y.-M., Gao W.-W. (2019). Effects of Maize Rotation on the Physicochemical Properties and Microbial Communities of American Ginseng Cultivated Soil. Sci. Rep..

[34] Li Y., Pu M., Cui Y., Gu J., Chen X., Wang L., Wu H., Yang Y., Wang C. (2023). Research on the Isolation and Identification of Black Spot Disease of Rosa Chinensis in Kunming, China. Sci. Rep..

[35] White T.J., Bruns T., Lee S., Taylor J. (1990). Amplification and Direct Sequencing of Fungal Ribosomal RNA Genes for Phylogenetics. PCR Protocols.

[36] Glass N.L., Donaldson G.C. (1995). Development of Primer Sets Designed for Use with the PCR to Amplify Conserved Genes from Filamentous Ascomycetes. Appl. Environ. Microbiol..

[37] Wang L., Zhuang W.Y. (2004). Designing Primer Sets for Amplification of Partial Calmodulin Genes from Penicillia. Mycosystema.

[38] Liu Y.J., Whelen S., Hall B.D. (1999). Phylogenetic Relationships among Ascomycetes: Evidence from an RNA Polymerase II Subunit. Mol. Biol. Evol..

[39] Thind K.S., Madan M. (1973). Effect of Various Carbon and Nitrogen Sources on the Growth and Sporulation of Claviceps Microcephala. Proc. Indian Acad. Sci.—Sect. B.

[40] Wei R., Wang R., Li Y., Yue M., Ding W. (2022). Identification, Biological Characteristics and Fungicide Sensitivity of *Colletotrichum* Species That Cause Anthracnose on *Anemarrhena asphodeloides* in China. J. Phytopathol..

[41] Wu W., Wang G., Li E., Tan S., Xu G., Huang X., Chen H., Liang Y., Li R., Qin J. (2024). Characterization and Fungicide Sensitivity of Phaeosphaeriopsis Obtusispora That Causes Marginal Leaf Blight in Agave Hybrid H.11648. JoF.

[42] Liu Y., Yang Y.-W., Miao Y.-H., Chen Q.-H., Wang T.-L., Liu D.-H., Huang B.-S. (2021). Identification and biological characteristics of southern blight causing root rot on three medicine plants of Iridaceae in Dabie Mountains. Zhongguo Zhong Yao Za Zhi.

