# Supplementary material for: First Report and Biological Characterization of Penicillium crustosum Causing Root Rot in Polygonatum kingianum (Yunnan, China)
Source: Plants (Basel). 2026 Jun 3;15(11):1739. doi: 10.3390/plants15111739 (PMC13259494; doi:10.3390/plants15111739)
Supplement: Supplementary file 1 [file plants-15-01739-s001.zip › Supplementary Fig S1-S4.pdf]

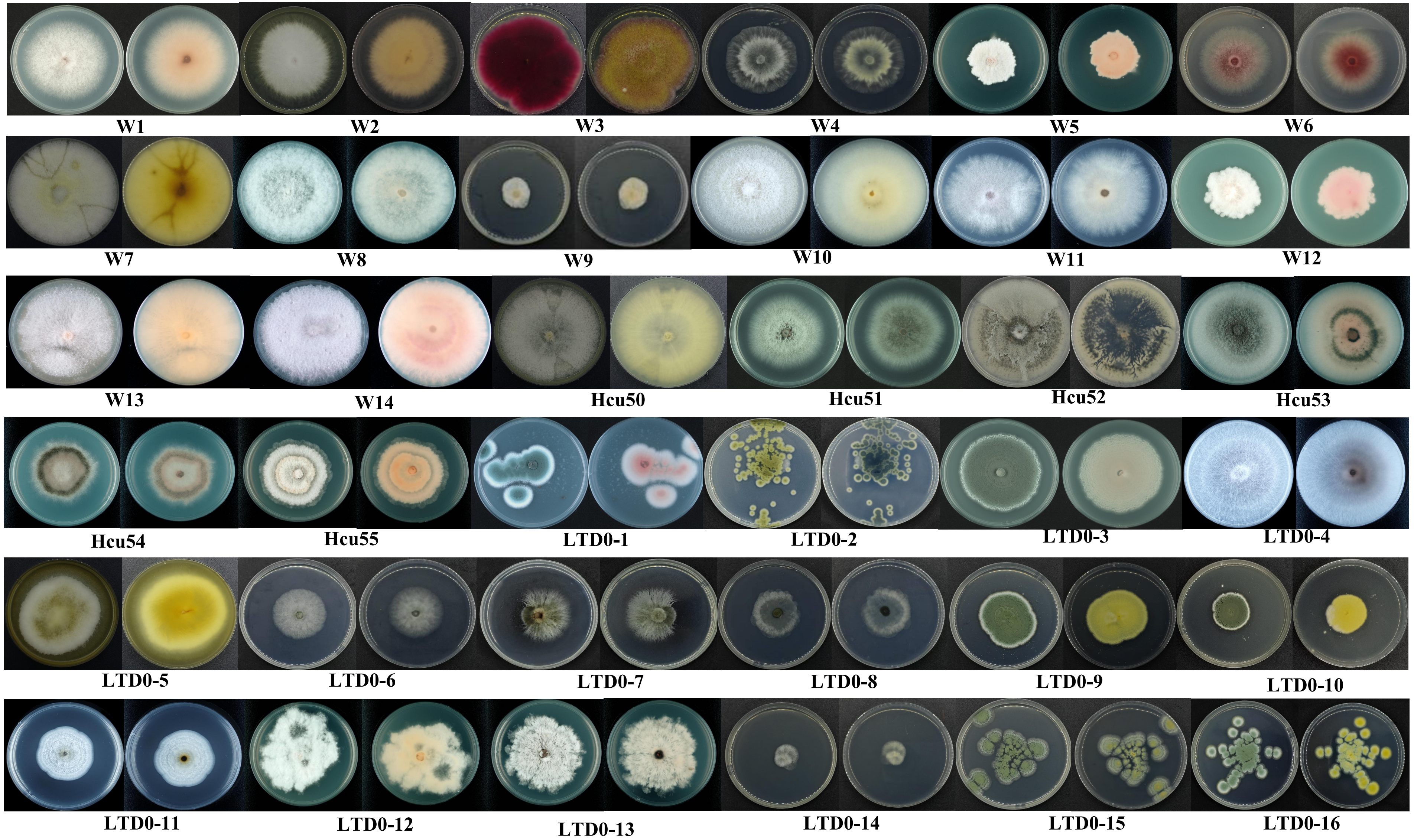

Supplementary Figure S1. Colony morphology of 36 fungal isolates causing root rot of *Polygonatum kingianum.*

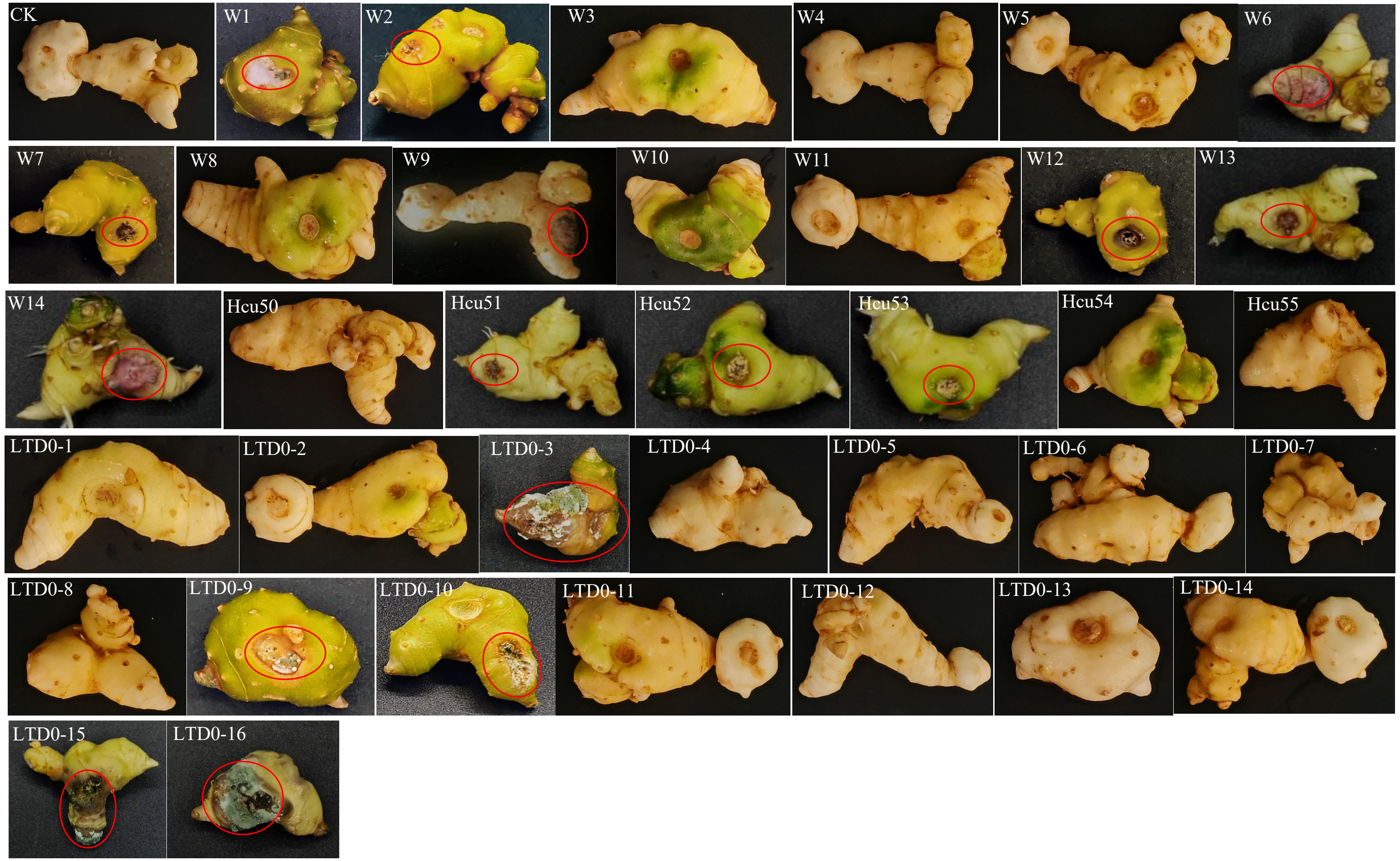

Supplementary Figure S2. Disease symptoms of *Polygonatum kingianum* rhizomes after inoculation with root rot pathogens.

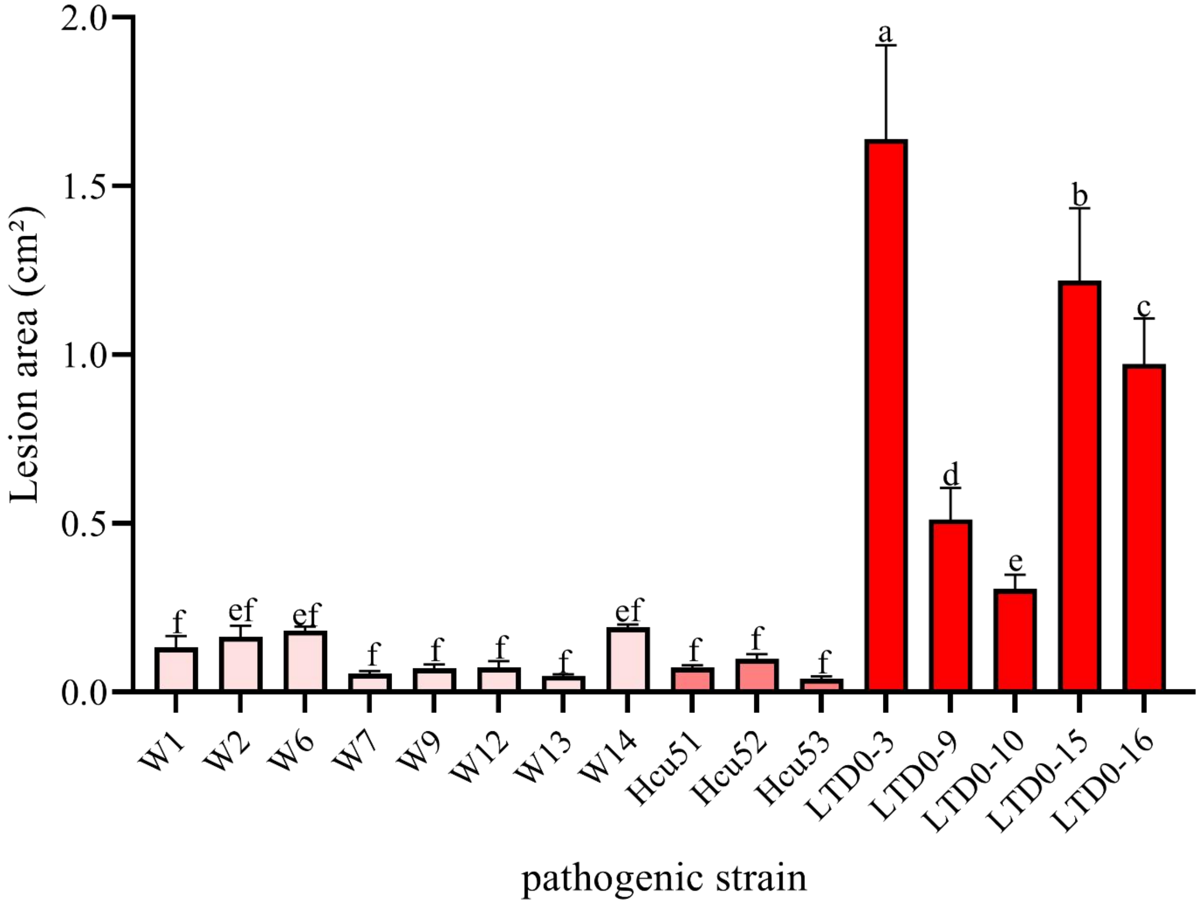

Supplementary Figure S3. Pathogenicity evaluation of root rot pathogens.

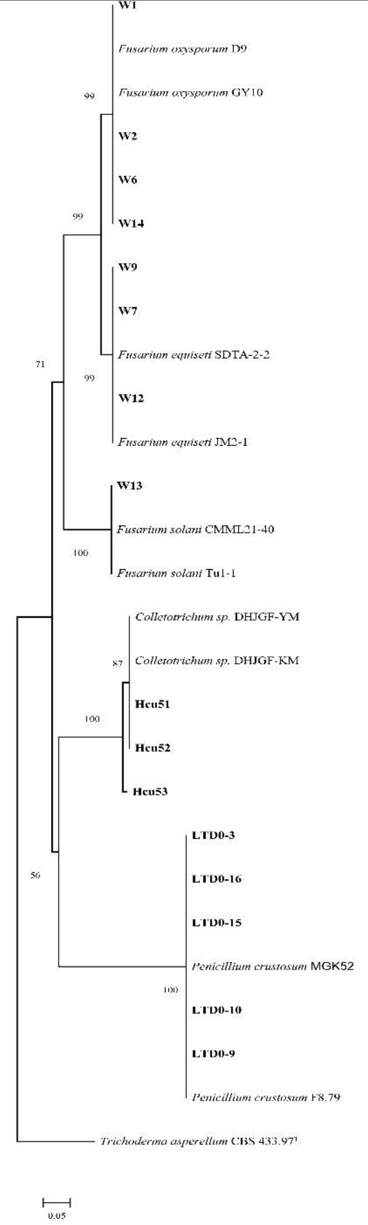

Supplementary Figure S4. Phylogenetic tree constructed using ITS sequences of pathogenic fungi causing root rot in *Polygonatum kingianum*. Note: The phylogenetic tree was constructed using the Maximum Likelihood (ML) method, with 1000 bootstrap replicates.
