# Supplementary figures and images for: First Report and Biological Characterization of Penicillium crustosum Causing Root Rot in Polygonatum kingianum (Yunnan, China)
Source: Plants (Basel). 2026 Jun 3;15(11):1739. doi: 10.3390/plants15111739 (PMC13259494; doi:10.3390/plants15111739)

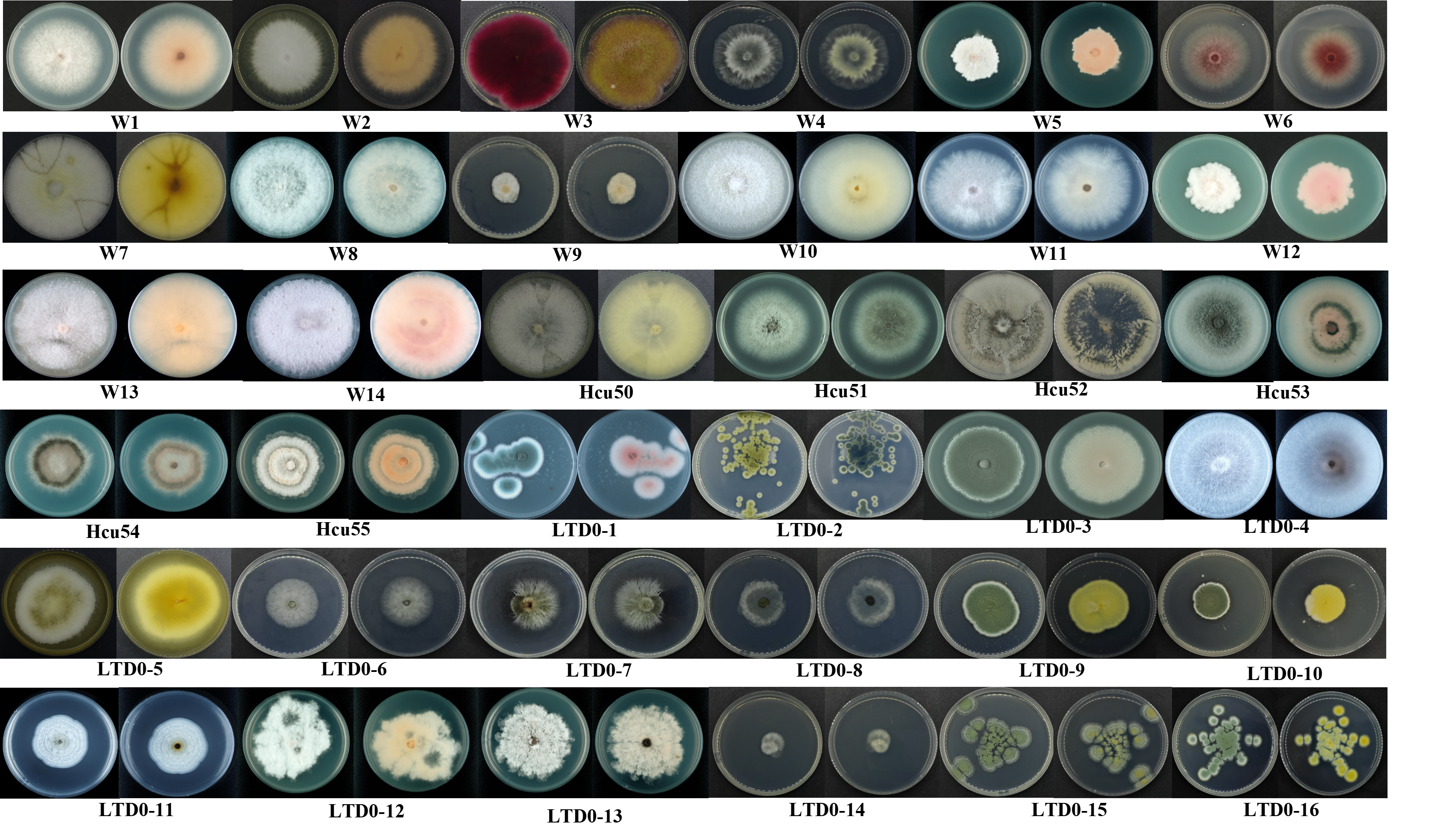

Supplement: Supplementary file 1 [file plants-15-01739-s001.zip › S1.tif]

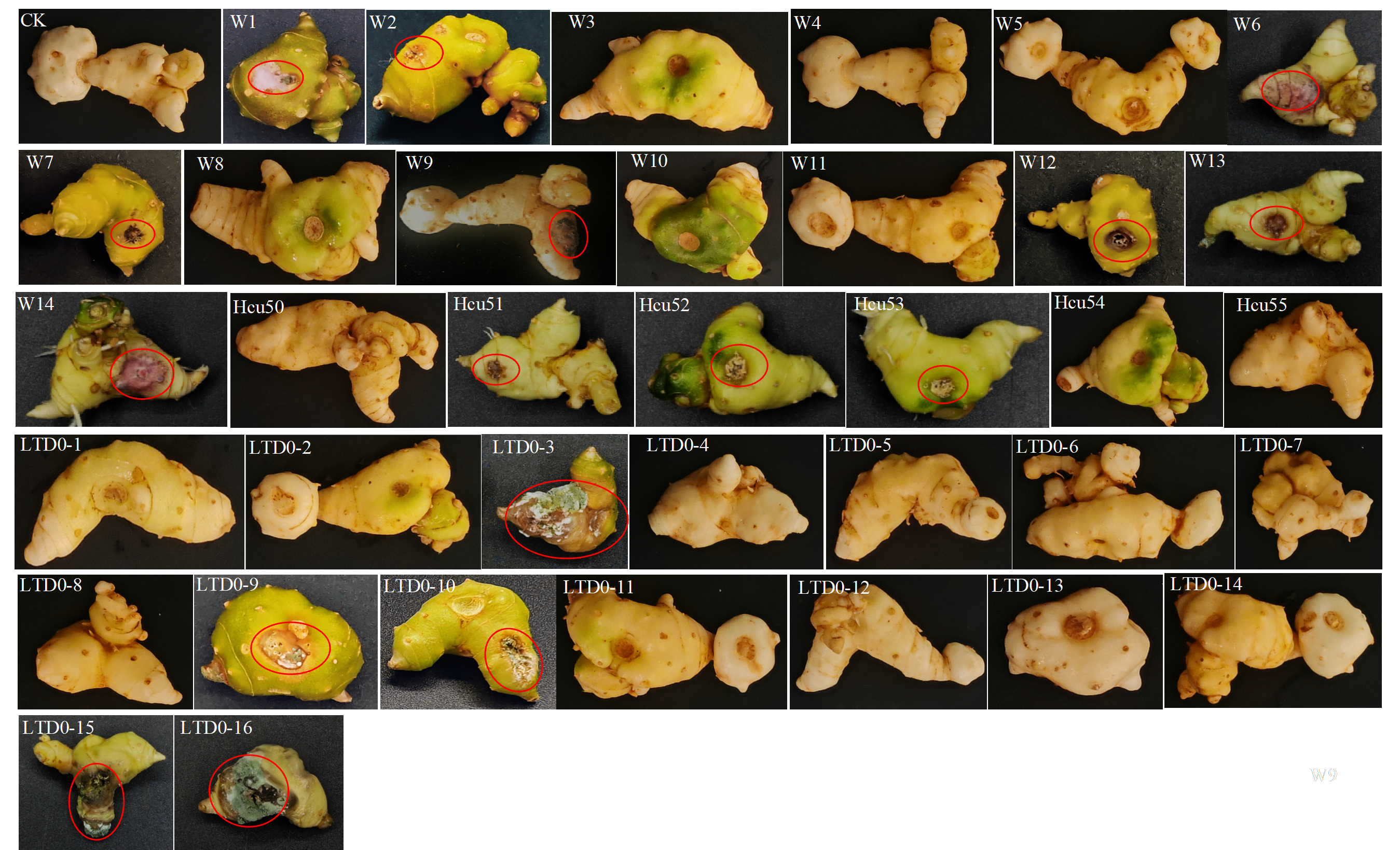

Supplement: Supplementary file 1 [file plants-15-01739-s001.zip › S2.tif]

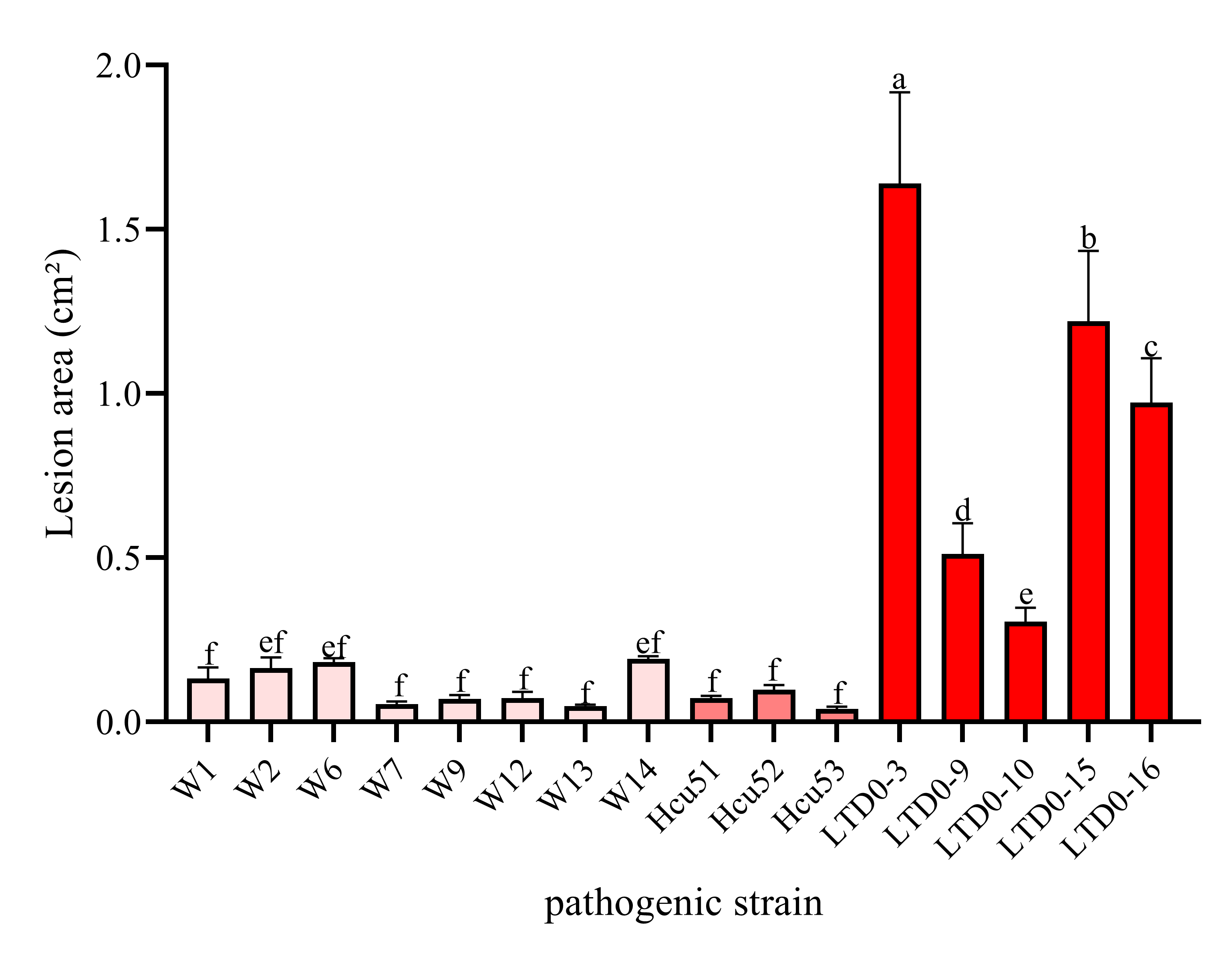

Supplement: Supplementary file 1 [file plants-15-01739-s001.zip › S3.tif]

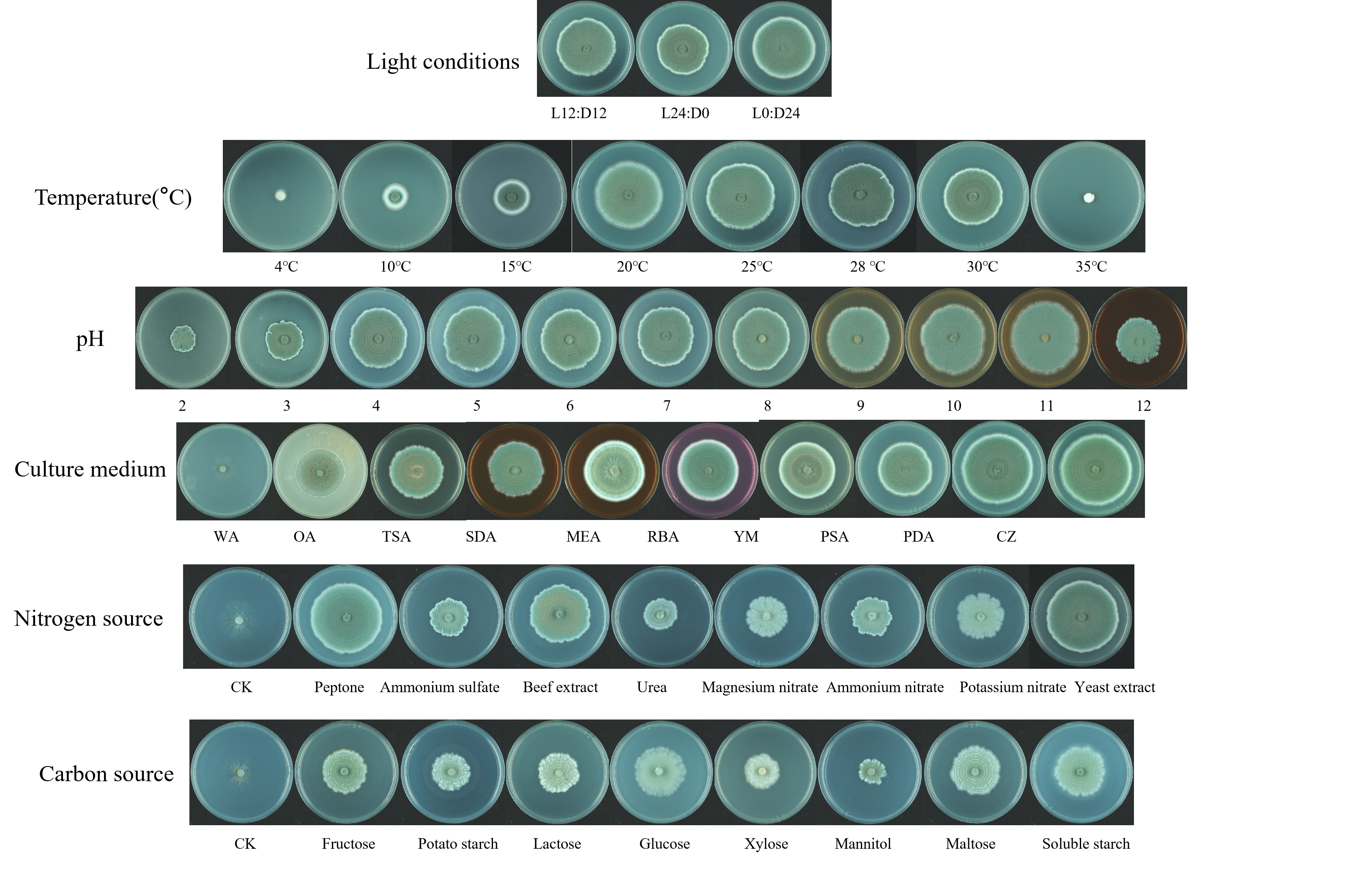

Supplement: Supplementary file 1 [file plants-15-01739-s001.zip › S5. Colony characteristics of the pathogen under diverse culture conditions.tif]
